# High Pretreatment D-Dimer Levels Correlate with Adverse Clinical Features and Predict Poor Survival in Patients with Natural Killer/T-Cell Lymphoma

**DOI:** 10.1371/journal.pone.0152842

**Published:** 2016-03-31

**Authors:** Xi-wen Bi, Liang Wang, Wen-wen Zhang, Peng Sun, Shu-mei Yan, Pan-pan Liu, Zhi-ming Li, Wen-qi Jiang

**Affiliations:** 1 Department of Medical Oncology, Sun Yat-sen University Cancer Center, Guangzhou, Guangdong, People’s Republic of China; 2 Department of Hematologic Oncology, Sun Yat-sen University Cancer Center, Guangzhou, Guangdong, People’s Republic of China; 3 Department of Radiation Oncology, Sun Yat-sen University Cancer Center, Guangzhou, Guangdong, People’s Republic of China; 4 Department of Pathology, Sun Yat-sen University Cancer Center, Guangzhou, Guangdong, People’s Republic of China; 5 State Key Laboratory of Oncology in South China, Collaborative Innovation Center for Cancer Medicine, Guangzhou, Guangdong, People’s Republic of China; The University of Hong Kong, CHINA

## Abstract

Pretreatment plasma D-dimer levels have been reported to predict survival in several types of malignancies. The aim of this study was to evaluate the prognostic value of D-dimer levels in patients with newly diagnosed natural killer/T-cell lymphoma (NKTCL). The cut-off value of D-dimer to predict survival was set as 1.2 μg/mL based on the receiver operating curve analysis. Patients with a D-dimer level ≥ 1.2 μg/mL had significantly more adverse clinical features, including poor performance status, advanced stage diseases, B symptoms, elevated serum lactic dehydrogenase levels, involvement of regional lymph nodes, more extranodal diseases, and higher International Prognostic Index and natural killer/T-cell lymphoma prognostic index scores. A D-dimer level ≥ 1.2 μg/mL was significantly associated with inferior 3-year overall survival (OS, 13.0 vs. 68.5%, *P* < 0.001). In the multivariate analysis, a D-dimer level ≥ 1.2 μg/mL remained an independent predictor for worse OS (HR: 3.13, 95% CI: 1.47–6.68, *P* = 0.003) after adjusting for other confounding prognostic factors. Among patients with Ann Arbor stage I-II diseases, those with a D-dimer level ≥ 1.2 μg/mL had a significantly worse survival than those with a D-dimer level < 1.2 μg/mL (3 year-OS: 76.2 vs. 22.2%, *P* < 0.001). Survival of early-stage patients with a high D-dimer level was similar to that of the advanced-stage patients. In conclusion, pretreatment plasma D-dimer level may serve as a simple but effective predictor of prognosis in patients with NKTCL.

## Introduction

Extranodal natural killer/T-cell lymphoma (NKTCL) is a relatively rare and unique subtype of lymphoid malignancy [1]. This disease shows an invasive biological behavior characterized by its destructive and ulcerative lesions (mostly in the nasal cavity), rapid clinical progression, and dismal outcomes. Extensive necrosis and inflammatory infiltrates are frequently observed in lesions under microscope and fever is a common clinical manifestation among the patients, indicating a strong association between NKTCL and inflammatory reactions [2,3]. Radiotherapy (RT) has been well established as the primary treatment for early-stage disease [4,5]. NKTCL was generally considered resistant to chemotherapy due to the expression of a multidrug-resistant gene [6,7], but novel regimens containing L-asparaginase (L-ASP) or pegaspargase have shown promising results [8–10]. Heterogeneous treatment response and prognosis have been observed in NKTCL. The International Prognostic Index (IPI) and natural killer/T-cell lymphoma prognostic index (NKPI) are the most commonly used models for prognostic stratification in NKTCL, but their accuracy remains controversial [11–13]. Therefore, it is important to explore more simple and accurate prognostic factors for risk stratification before treatment.

D-dimer is produced when cross-linked fibrin polymer is degraded by plasmin during the process of fibrinolysis, which is up regulated as a compensation mechanism for fibrin clot formation during coagulation. An elevated level of D-dimer is a useful biomarker for the activation of hemostasis and fibrinolysis [14]. In previous studies, elevated levels of D-dimer have been reported to correlate with adverse prognosis in several types of malignancies, including breast [15], colorectal [16,17], lung [18,19], and gastric cancers [20]. The mechanism underlying the prognostic value of D-dimer in cancer patients is not fully understood. However, it has been reported that D-dimer can promote tumor cell proliferation, adhesion, angiogenesis, and ultimately contributes to the growth and metastasis of malignancies [21]. On the other hand, the coagulation pathway can be activated by tumor cells via releasing tissue factors and promoting endothelial cells to produce procoagulants, resulting in compensatory activation of fibrinolysis and D-dimer production [22,23]. So far, the clinical significance of D-dimer in NKTCL has not been reported. The purpose of this study is to analyze the correlation between pre-treatment plasma D-dimer levels and clinical features as well as survival in patients with newly diagnosed NKTCL.

## Materials and Methods

### Patient selection

The medical records of patients with newly diagnosed NKTCL at Sun Yat-sen University Cancer Center between 2007 and 2013 were reviewed. The inclusion criteria included: (1) diagnosis of NKTCL according to the World Health Organization (WHO) classification of lymphomas [2,3]; (2) available results of plasma D-dimer level within seven days before initial treatment; and (3) complete follow-up data. The exclusion criteria were: (1) known active infection when the baseline D-dimer levels were taken; (2) a history of venous or arterial thromboembolism or anticoagulant treatment within 3 months before treatment; (3) pregnancy, stroke, or neurosurgery within 6 months before treatment; and (4) known congenital coagulative abnormality. A total of 84 patients were identified and included in the analysis. Written informed consent for collecting medical information was obtained from all patients at their first visits. The ethics committee of Sun Yat-sen University Cancer Center approved this study (Approval No. B2015-055-12). All authors had access to identifying patient information from the medical records of the 84 previously untreated patients with NKTCL. All procedures performed in studies involving human participants were in accordance with the ethical standards of the institutional and/or national research committee and with the 1964 Helsinki declaration and its later amendments or comparable ethical standards.

### Staging and D-dimer evaluations

All patients underwent a clinical staging evaluation before treatment, including a medical history and physical examination; complete blood count; serum biochemistry, including the serum lactic dehydrogenase (LDH) levels; computed tomography and/or magnetic resonance of the head and neck; computed tomography of the chest, abdomen, and pelvis; and bone marrow aspiration. The patients were staged according to the modified Ann Arbor staging system [24]. The International Prognostic Index (IPI, including age, performance status, stage of disease, LDH level, and number of extranodal lesions) and the natural killer/T-cell lymphoma prognostic index (NKPI, including stage of disease, the involvement of regional lymph nodes, B symptoms, and LDH level) were calculated for all patients [11,13].

Fasting venous blood samples were collected within 7 days before initial treatment. Samples were centrifuged to obtain platelet-free plasma. D-dimer levels were measured by a latex-enhanced immunoturbidimetric assay (Sekisui Medical Co., Ltd., Tokyo, Japan) on a Sysmex CA 7000 (Sysmex Co., Kobe, Japan) analyzer according to the manufacturer’s instructions.

### Treatment modality and follow-up

The treatment modality has been described previously [25,26]. Patients with early stage disease received induction chemotherapy followed by involved-field radiotherapy (IFRT). Patients with advanced disease received chemotherapy as the primary treatment, and IFRT could be delivered subsequently as consolidation, palliative, or salvage therapy according to the physician’s clinical judgment. Chemotherapy regimens varied over the study period and were categorized as an asparaginase (ASP)-containing or ASP-absent regimen, depending on whether L-ASP or pegaspargase was used [25,26]. The radiation techniques (including the dose and field) have been previously described [27]. Among the 84 patients included in this study, 51 (60.7%) received IFRT with or without chemotherapy, 28 (33.3%) received chemotherapy alone, and 5 (6.0%) received best supportive care alone. Fifty patients (66.7%) received ASP-absent chemotherapy while 25 (33.3%) received ASP-containing chemotherapy. The median number of chemotherapy cycles was 3 (range 0–8). The median radiation dose was 56.0 Gy (range 40–74 Gy).

Patients were followed every 3 months for the first 2 years after initial treatment, every 6 months for the next 3 years, and annually thereafter. The follow-up evaluation was carried out as previously reported [28].

### Statistical analysis

The overall survival (OS) was measured from the date of diagnosis to the date of death due to any cause or the date of the most recent follow-up. The survival data were calculated using the Kaplan-Meier method and compared using the log-rank test. The optimal cut-off value of D-dimer levels for predicting survival was determined using the receiver operating curve (ROC) analysis. Continuous variables were compared using the Mann-Whitney U-test, and categorical variables were compared using the Chi-squared test or the Fisher exact test. The Spearman correlation test was utilized to examine the association between D-dimer levels and other clinical variables. The Cox proportional hazard model was used for a univariate screen of all potential predictors of survival. Variables with statistical and clinical significance were included in the multivariate analysis using a stepwise forward Cox regression model. Results were considered statistically significant with a two-sided P value < 0.05. The statistical analysis was performed using SPSS version 17.0 software (SPSS A, Inc., Chicago, IL, USA).

## Results

### Determination of the cut-off value of plasma D-dimer levels

**Fig 1:**
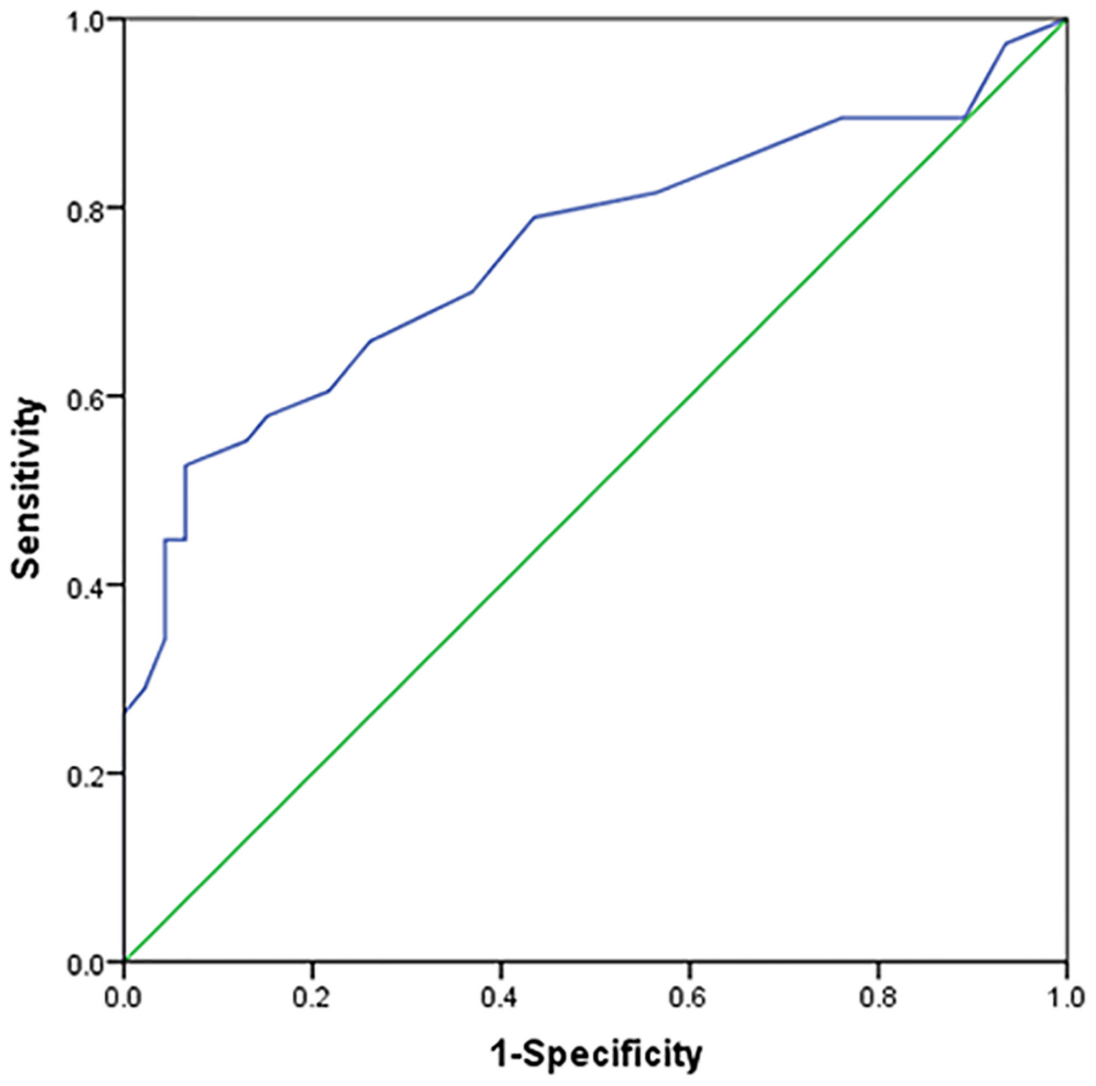
The receiver operating curve (ROC) analysis of pretreatment plasma D-dimer levels. The optimal cut-off value for D-dimer levels to predict mortality is 1.2μg/mL (sensitivity = 52.6%, specificity = 93.5%, accuracy = 75.0%).

For the entire cohort, the median value of plasma D-dimer was 0.7 μg/mL (range 0.1–55.4 μg/mL). The optimal cut-off value of D-dimer to predict survival was 1.2 μg/mL based on the ROC analysis (Fig 1). The area under curve (AUC) was 0.753 (95% CI: 0.644–0.863, *P* < 0.001). At a cut-off value of 1.2 μg/mL, the D-dimer level had a sensitivity of 52.6%, a specificity of 93.5% and an accuracy of 75.0% to predict mortality.

### Plasma D-dimer levels and clinical characteristics

Baseline clinical characteristics of the cohort are presented and compared between patients with a low (< 1.2 μg/mL) or high (≥ 1.2 μg/mL) D-dimer in Table 1. Patients with a D-dimer level ≥ 1.2 μg/mL presented with significantly more adverse clinical features, including significantly worse Eastern Cooperative Oncology Group (ECOG) performance scores (ECOG ≥ 2: 69.6 vs. 6.6%, *P* < 0.001), more advanced diseases (stage III-IV: 60.9 vs. 11.5%, *P* < 0.001), B symptoms (91.3 vs. 55.7%, *P* = 0.002), elevated LDH levels (73.9 vs. 21.3%, *P* < 0.001), involvement of regional lymph nodes (65.2 vs. 31.1%, *P* = 0.005), more extranodal diseases (≥ 2 sites: 47.8 vs. 11.5%, *P* < 0.001), and higher IPI scores (P < 0.001) and NKPI scores (P < 0.001). Treatment modalities also differed between those two groups. There were significantly fewer patients who received radiotherapy in the group with a D-dimer level ≥ 1.2 μg/mL, compared with those with a D-dimer level < 1.2 μg/mL (21.7 vs. 75.4%, *P* < 0.001). Chemotherapeutic regimens were comparable between the two groups (*P* = 0.566). Spearman correlation tests further confirmed that plasma D-dimer levels correlated with performance score, Ann Arbor stage, B symptoms, LDH levels, involvement of regional lymph nodes, number of extranodal diseases, treatment modalities, IPI score, and NKPI score (Table 2).

**Table 1:** The clinical characteristics of patients with NK/T-cell lymphoma.

Parameters ^a^ *n* (%)	Total	D-Dimer < 1.2 μg/mL	D-Dimer ≥ 1.2 μg/mL	*P* value
Overall	84 (100)	61 (100)	23 (100)	
Male gender	57 (67.9)	40 (65.6)	17 (73.9)	0.466
Age (years)	43 (11–77)	41 (16–77)	49 (11–75)	0.140
Age ≥ 60 years	15 (17.9)	10 (16.3)	5 (21.7)	0.568
ECOG score ≥ 2	20 (23.8)	4 (6.6)	16 (69.6)	< 0.001
Ann Arbor stage				
I-II	63 (75.0)	54 (88.5)	9 (39.1)	< 0.001
III-IV	21 (25.0)	7 (11.5)	14 (60.9)	
B symptoms	55 (65.5)	34 (55.7)	21 (91.3)	0.002
Elevated LDH	30 (35.7)	13 (21.3)	17 (73.9)	< 0.001
Involvement of regional lymph nodes	34 (40.5)	19 (31.1)	15 (65.2)	0.005
Extranodal sites ≥ 2	18 (21.4)	7 (11.5)	11 (47.8)	< 0.001
IPI score				
Low risk (0–1)	61 (72.6)	53 (86.9)	8 (34.8)	< 0.001
Intermediate risk (2–3)	9 (10.7)	6 (9.8)	3 (13.0)	
High risk (4–5)	14 (16.7)	2 (3.3)	12 (52.2)	
NKPI score				
Low risk (0)	19 (22.6)	18 (29.5)	1 (4.3)	< 0.001
Intermediate risk (1–2)	40 (47.6)	34 (55.7)	6 (26.1)	
High risk (3–4)	25 (29.8)	9 (14.8)	16 (69.6)	
Treatment modalities				
Chemotherapy alone	28 (33.3)	15 (24.6)	13 (56.5)	< 0.001
RT ± chemotherapy	51 (60.7)	46 (75.4)	5 (21.7)	
Best supportive care alone	5 (6.0)	0 (0.0)	5 (21.7)	
Chemotherapeutic regimen				
Asparaginase-containing	25 (33.3)	18 (31.6)	7 (38.9)	0.566
Asparaginase-absent	50 (66.7)	39 (68.4)	11 (61.1)	

ECOG: Eastern Cooperative Oncology Group; IPI: International Prognostic Index; LDH: lactate dehydrogenase; NKPI: natural killer/T-cell lymphoma prognostic index; RT: radiotherapy.

^a^ Continuous variables are presented as medians (range), and categorical variables are shown as frequencies and percentages.

**Table 2:** Correlation between pretreatment plasma D-dimer levels and clinicopathological factors in patients with NK/T-cell lymphoma.

Parameters	Spearman correlation coefficient	*P* value
Sex (male/female)	0.031	0.783
Age	-0.025	0.822
ECOG score (0/1/2)	0.599	< 0.001
Ann Arbor stage (I/II/III/IV)	0.372	< 0.001
B symptoms (yes/no)	0.399	< 0.001
LDH level	0.495	< 0.001
Involvement of regional lymph nodes (yes/no)	0.254	0.020
Extranodal sites(0-1/ ≥ 2)	0.295	0.006
IPI score (0/1/2/3/4/5)	0.479	< 0.001
NKPI score (0/1/2/3/4)	0.526	< 0.001
Treatment modalities (RT/no RT)	-0.405	< 0.001
Chemotherapeutic regimen (ASP/no ASP)	0.009	0.942

ASP: asparaginase; ECOG: Eastern Cooperative Oncology Group; IPI: International Prognostic Index; LDH: lactate dehydrogenase; NKPI: natural killer/T-cell lymphoma prognostic index; RT: radiotherapy.

### Plasma D-dimer levels and survival

**Fig 2:**
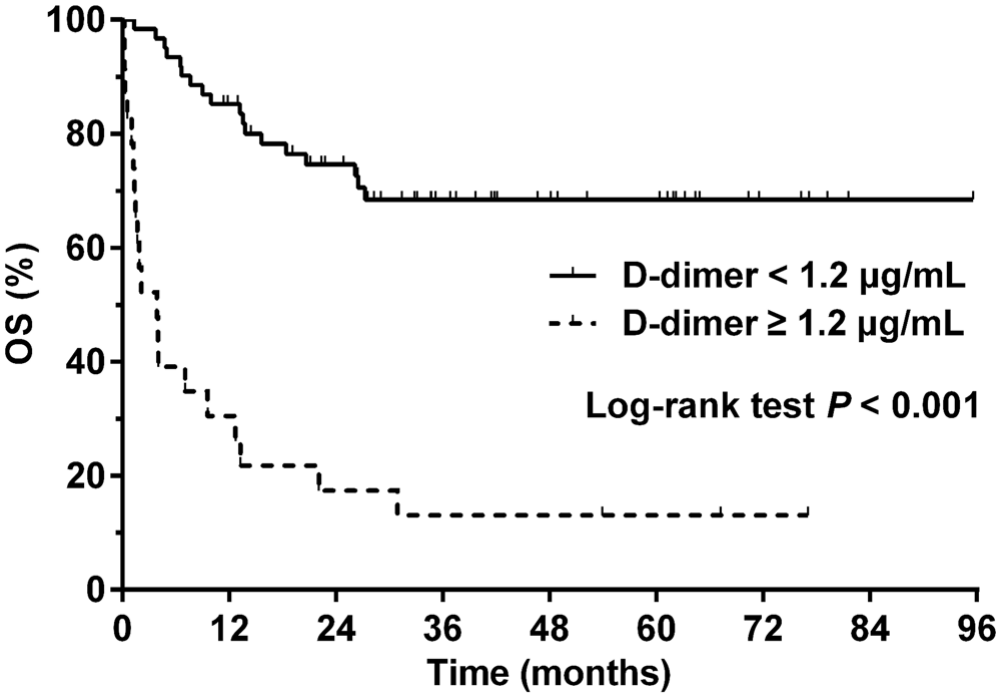
Overall survival (OS) in patients with high (≥ 1.2 μg/mL, dashed line) or low (<1.2 μg/mL, solid line) pretreatment plasma D-dimer levels.

The median follow-up period for surviving patients was 41.7 months (range 11.4–95.6 months). A total of 38 (45.2%) patients died from lymphoma (*n* = 36) or treatment-related complications (*n* = 2). The 3-year OS for the entire cohort was 52.8% and the median survival was not reached. The median plasma D-dimer level of patients who deceased was significantly higher than that of those who survived (1.4 vs. 0.5 μg/mL, *P* < 0.001). A higher D-dimer level (≥ 1.2 μg/mL) was significantly associated with inferior 3-year OS (13.0 vs. 68.5%, *P* < 0.001, Fig 2). Two patients in the high D-dimer group experienced symptomatic venous thromboembolism (VTE) associated with central venous catheters, and they were alive without tumor at 24.8 and 41.5 months after diagnosis, respectively.

Results of the univariate screening for prognostic factors for OS are presented in Table 3. The hazard ratio (HR) for the OS of the patients with a D-dimer level ≥ 1.2 μg/mL was 6.38 [95% confidence interval (CI): 3.34–12.20, *P* < 0.001] compared with those with a D-dimer level < 1.2 μg/mL. Other parameters that predicted significantly shorter OS included an age of > 60 years, an ECOG score of ≥ 2, Ann Arbor stage III-IV, B symptoms, an elevated LDH level, involvement of ≥ 2 extranodal sites, an intermediate- or high-risk IPI or NKPI score, and treatment without radiotherapy. The parameters of survival significance in univariate analysis were included in the multivariate analysis, except the IPI and NKPI because they consisted of some other parameters that were already included in the model. In the multivariate Cox regression model, a D-dimer level ≥ 1.2 μg/mL remained an independent predictor for worse OS (HR: 3.13, 95% CI: 1.47–6.68, *P* = 0.003, Table 3). Other independent prognostic factors were age > 60 years (HR: 6.11, 95% CI: 2.65–14.12, *P* < 0.001), two or more extranodal diseases (HR: 2.25, 95% CI: 1.01–5.02, *P* = 0.048), and treatment without radiotherapy (HR: 9.87, 95% CI: 4.14–23.57, *P* < 0.001).

**Table 3:** Univariate and multivariate analysis of prognostic factors for overall survival in patients with NK/T-cell lymphoma.

	Univariate analysis		Multivariate analysis	
Variable	HR (95% CI)	*P* value	HR (95% CI)	*P* value
Gender (female vs. male)	0.70 (0.34–1.45)	0.337		
Age (> 60 vs. ≤ 60 years)	2.45 (1.21–4.94)	0.013	6.11 (2.65–14.12)	<0.001
ECOG score (≥ 2 vs. 0–1)	4.73 (2.48–9.03)	< 0.001		
Stage (III-IV vs. I-II)	5.46 (2.84–10.50)	< 0.001		
B symptoms (yes vs. no)	3.06 (1.35–6.96)	0.008		
LDH (elevated vs. normal)	4.06 (2.12–7.76)	< 0.001		
Involvement of regional lymph nodes (yes vs. no)	2.82 (1.47–5.40)	0.002		
Extranodal sites (≥ 2 vs. 0–1)	3.87 (2.00–7.47)	< 0.001	2.25 (1.01–5.02)	0.048
D-Dimer (≥ 1.2 vs. < 1.2 μg/mL)	6.38 (3.34–12.20)	< 0.001	3.13 (1.47–6.68)	0.003
IPI (ref: 0–1)				
IPI (2–3)	3.33 (1.39–7.98)	0.007		
IPI (4–5)	6.87 (3.31–14.26)	< 0.001		
NKPI (ref: 0)				
NKPI (1–2)	10.08(1.34–75.78)	0.025		
NKPI (3–4)	29.00 (3.88–216.8)	0.001		
Treatment modalities (no RT vs. RT)	8.76 (4.11–18.64)	< 0.001	9.87 (4.14–23.57)	< 0.001
Chemotherapeutic regimen (ASP vs. no ASP)	1.22 (0.57–2.63)	0.606		

ASP: asparaginase; CI: confidence interval; ECOG: Eastern Cooperative Oncology Group; HR: hazard ratio; IPI: International Prognostic Index; LDH: lactate dehydrogenase; NKPI: natural killer/T-cell lymphoma prognostic index; RT: radiotherapy.

### Prognostic significance of D-dimer levels according to Ann Arbor stage

**Fig 3:**
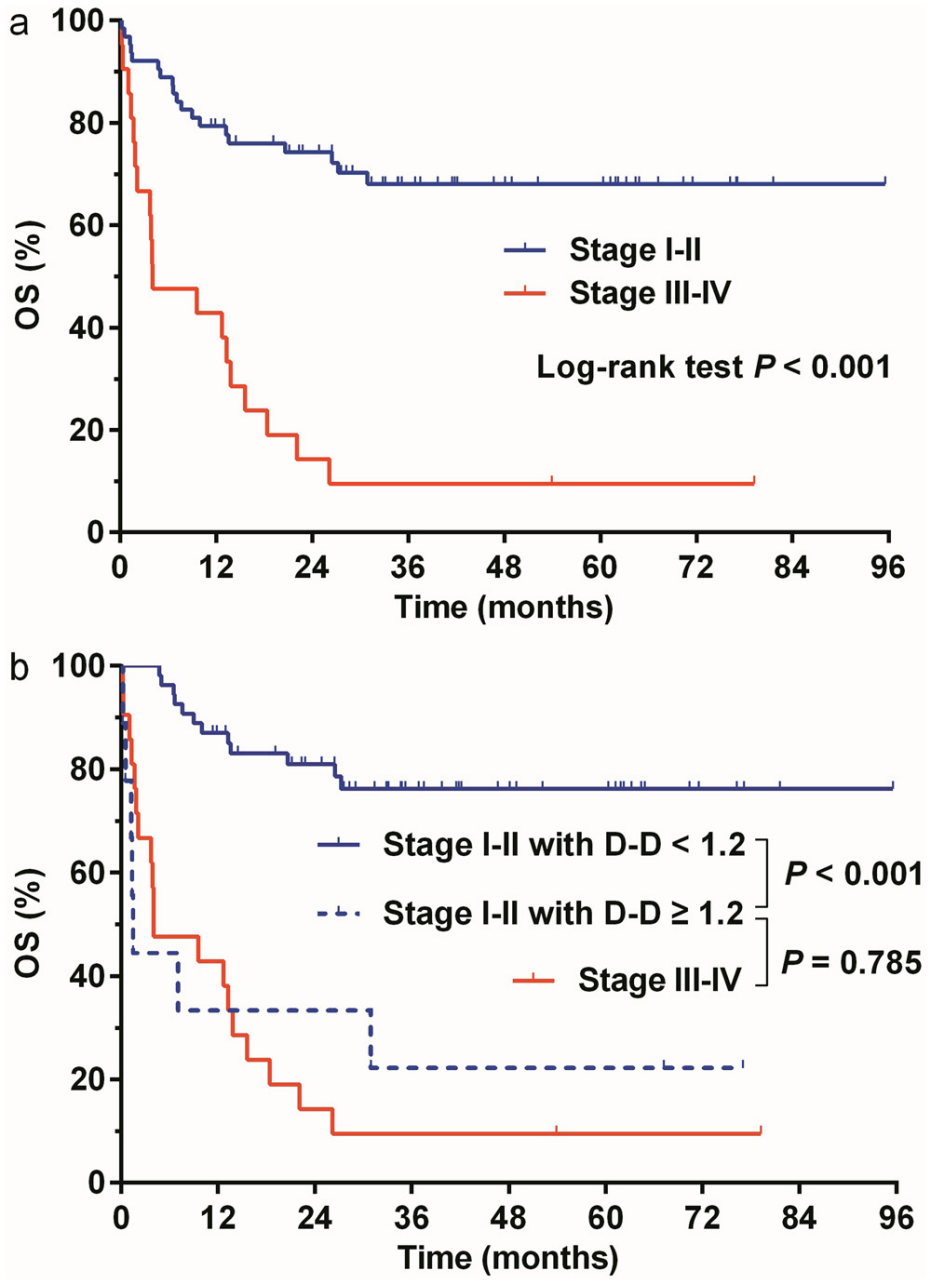
Overall survival (OS) according to Ann Arbor stage and D-dimer (D-D) levels. (a) OS differed significantly (*P* < 0.001) between patients with Ann Arbor stage I-II (blue line) or III-IV (red line) disease. (b) Among patients with stage I-II disease, OS was significantly worse in those with D-D levels of ≥ 1.2μg/mL (dashed blue line) compared with those with D-D levels of < 1.2μg/mL (solid blue line, *P <* 0.001). OS were similar between stage I-II patients with D-D levels of ≥ 1.2μg/mL (dashed blue line) and stage III-IV patients (solid red line, *P* = 0.785).

We further evaluated the prognostic value of elevated D-dimer levels in early-stage and advanced-stage patients, respectively. Patients with Ann Arbor stage I-II (early-stage) disease showed a substantially better 3-year OS than those with stage III-IV (advanced-stage) disease (68.0 vs. 9.5%, *P* < 0.001, Fig 3a). However, 9 out of the 63 (14.3%) early-stage patients had a D-dimer level ≥ 1.2 μg/mL, and their survival was significantly worse than that of the remaining 54 patients with a D-dimer level < 1.2 μg/mL (3 year-OS: 76.2 vs. 22.2%, *P* < 0.001, Fig 3b). In fact, survival of early-stage patients with a D-dimer level ≥ 1.2 μg/mL was similar to that of the advanced-stage patients (3 year-OS: 22.2 vs. 9.5%, *P* = 0.785, Fig 3b). However, D-dimer levels had no significant discriminative effect among patients with advanced-stage disease (3-year OS was 14.3% for the D-dimer < 1.2 μg/mL group and 7.1% for the D-dimer ≥ 1.2 μg/mL group, respectively, *P* = 0.145).

## Discussion

In this study, we retrospectively reported the clinical and prognostic implications of pretreatment plasma D-dimer levels in patients with newly diagnosed NKTCL. We selected 1.2 μg/mL as the cut-off value for D-dimer according to the ROC analysis. Results showed that: (1) high levels of pretreatment D-dimer were associated with significantly more adverse clinical characteristics, including poor performance status, advanced diseases, elevated LDH levels, and higher IPI and NKPI scores, etc; (2) high levels of D-dimer were associated with substantially worse survival and remained an independent adverse prognostic factor in the multivariate analysis; (3) high levels of D-dimer could indentify high-risk individuals with significantly poor survival from those with early-stage disease.

Previous studies have demonstrated that a high level of plasma D-dimer correlated with poor prognosis in several types of solid malignancies [15–20], but its prognostic value has been rarely reported in lymphoid malignancies. In a large prospective cohort study, an elevated D-dimer level was associated with increased mortality risk in patients with lymphoma. However, further analysis by different lymphoma subtypes was not carried in this study [29]. In the present study, we explored the prognostic value of plasma D-dimer levels in a specific subtype of lymphoma, the NKTCL. Our study demonstrated for the first time that high levels of pretreatment D-dimer levels correlated with adverse clinical profiles and inferior survival in NKTCL patients. D-dimer, as a routinely used and easily measured marker, may serve as a useful prognostic predictor in clinical practice for this relatively rare malignancy.

The exact mechanisms underlying the association between the D-dimer levels and cancer survival remain to be investigated. Elevated D-dimer levels have been reported to be associated with a heavier tumor burden in solid malignancies, including advanced tumor stage, regional lymph node involvement, and distant metastasis [30–32]. D-dimer has also been reported to promote tumor cell proliferation, adhesion, and metastasis [21]. In our cohort of NKTCL, patients with high D-dimer levels presented with more advanced stage disease, more regional lymph node involvement, and more extranodal diseases (Table 1), which were consistent with the results observed in previous studies of solid malignancies. As expected, those clinical parameters indicative of a heavier tumor burden were associated with an increased mortality in our study. However, the inferior survival observed in NKTCL patients with elevated D-dimer levels was independent of those parameters in the multivariate analysis. Therefore, the adverse prognostic effect of elevated D-dimer levels cannot be solely explained by the high tumor burden. In addition, elevated D-dimer levels were found to predict the occurrence of VTE, which is more frequently observed in cancer patients and poses a high risk of mortality [14,18]. However, only two patients with a high D-dimer level in our cohort experienced symptomatic VTE and they had relatively good prognosis, indicating that VTE might not be the reason for the dismal survival in patients with high levels of D-dimer. Another possible explanation is that tumor cells can activate the coagulation cascade by releasing tissue factors and cytokines, resulting in subsequent activation of fibrinolysis and D-dimer accumulation [22,23]. As several cytokines, including tumor necrosis factor and interleukins, have been correlated with disease status and survival in NKTCL patients [33–36], the association between those molecules and plasma D-dimer levels in NKTCL may require further exploration.

Treatment modality is an important prognostic factor in almost all kinds of malignancies. In our cohort, there were significantly fewer patients who received radiotherapy in the group with a D-dimer level ≥ 1.2 μg/mL, compared with those with a D-dimer level < 1.2 μg/mL (21.7 vs. 75.4%, *P* < 0.001). Because D-dimer itself is not a determinative factor for treatment planning in our clinical practice, the difference in treatment modality resulted mainly from the adverse clinical features observed in the high D-dimer group, including significantly worse performance status and more advanced diseases. In consistency with previous studies [4, 25, 26], radiotherapy ± chemotherapy was associated with improved survival in the univariate and multivariate analysis compared with chemotherapy alone. However, D-dimer still remained an independent prognostic factor when included together with treatment modalities in the Cox regression model. Therefore, the prognostic power of D-dimer levels does not depend on treatment modalities.

The Ann Arbor staging system for lymphoma was first proposed in 1971 [37] and then modified in 1988 [24], which was before NKTCL was recognized as an independent pathologic entity. The major limitation of this staging system is that it takes no account of the heterogeneous pathologic subtypes and anatomic sites of lymphoma. Previous studies have demonstrated that a proportion of the so-called "early-stage" (Ann Arbor stage I-II) NKTCL patients responded poorly to the first-line treatment and had dismal survival, calling for an effective parameter to identify high-risk individuals from early-stage patients [4,38,39]. According to our results, the early-stage NKTCL patients with a D-dimer level ≥ 1.2 μg/mL had a substantially worse survival than those with a low D-dimer level (Fig 3b). Although those patients with high D-dimer levels were still classified as having “early-stage” disease in the Ann Arbor system, their survival was not statistically different from that of the advanced-stage patients (Fig 3b). These findings have very important clinical implications that early-stage NKTCL patients with high D-dimer levels should be treated as high-risk individuals and may require more intensive therapy. The results also suggest that the current Ann Arbor staging system for NKTCL requires further improvement, and a simple serum biomarker may serve as a useful supplement to it.

There are several limitations in our study due to its retrospective nature. D-dimer levels were measured only once at baseline, and data on their dynamic changes during the treatment and follow-up period were unavailable. In addition, only symptomatic VTE was reported in this study because a routine screen for occult thrombus was not part of the clinical practice. Asymptomatic VTE might present in some patients (especially those with high D-dimer levels) and elicit adverse effects on their disease course and survival.

## Conclusions

A high level of pretreatment plasma D-dimer could serve as a simple but effective predictor of adverse survival in patients with newly diagnosed NKTCL. In addition, elevated levels of D-dimer could further identify high-risk patients from those with Ann Arbor early-stage diseases. The findings of this single-center study require further validation in other cohorts with a prospective study design.

## Supporting Information

S1 Table Raw data of the study. (XLSX)

S1 Text Ethical approval. (PDF)
