# Supplementary material for: High Pretreatment D-Dimer Levels Correlate with Adverse Clinical Features and Predict Poor Survival in Patients with Natural Killer/T-Cell Lymphoma
Source: PLoS One. 2016 Mar 31;11(3):e0152842. doi: 10.1371/journal.pone.0152842 (PMC4816543; doi:10.1371/journal.pone.0152842)
Supplement: S1 Text — (PDF) [file pone.0152842.s002.pdf]

Sun Yat-sen University Cancer Center IRB

中山大学肿瘤防治中心伦理委员会

审批号/ Approval No.: B2015-055-12 **审批日期/Approval Date:** 2015/11/10

地址：广州市越秀区东风东路 651 号邮编：510060

电话/ Tel: 87343135 传真/ Fax: 87343009

Address: No. 651, Dongfeng East Road, Guangzhou, 510060, P.R.China

Protocol title: Clinical and prognostic significance of pretreatment plasma D-dimer levels in patients with newly-diagnosed NK/T-cell lymphoma

Protocol version & Date: Version 1.0, 2015-10-03

Informed consent version & Date: Version 1.0, 2015-10-03

Study site & Principle Investigator: Sun Yat-sen University Cancer Center & Wen-qi Jiang

Two IRB members of SYSUCC have expedited review the proposal on 2015/11/10, in accordance to ICH GCP guidelines, government regulations and laws, and agreed to approve this protocol.

Conditions: 1. Do not deviate from, or make changes to study protocol without prior written IRB approval, except when it is necessary to eliminate immediate hazards to research subjects or when the change involves only logistical or administrative issues; 2. Report the following to SYSUCC IRB: (1) study protocol or consent document change, (2) serious adverse event, (3) study progress, and (4) new information that may be relevant to a subject's willingness to continue participation in the study; 3. Report study progress to SYSUCC IRB at a 12-monthly interval until study closure.

Peng Wang qing

Peng Wang-qing

IRB Chairman

Sun Yat-sen University Cancer Center

2015/11/10
